# Image Quality and Dose Comparison of Single-Energy CT (SECT) and Dual-Energy CT (DECT)

**DOI:** 10.1155/2020/1403957

**Published:** 2020-04-20

**Authors:** Ramin Ghasemi Shayan, Maryam Oladghaffari, Fakhrosadat Sajjadian, Mona Fazel Ghaziyani

**Affiliations:** Radiology Department, Tabriz University of Medical Sciences, Tabriz 51368, Iran

## Abstract

CT and its comprehensive usage have become one of the most indispensable components in medical field especially in the diagnosis of several diseases. SECT and DECT have developed CT diagnostic potentials in several means. In this review article we have discussed the basic principles of single-energy and dual-energy computed tomography and their important physical differences which can cause better diagnostic evaluation. Moreover, different organs diagnostic evaluations through single-energy and dual-energy computed tomography have been discussed. Conventional or single-energy CT (SECT) uses a single polychromatic X-ray beam (ranging from 70 to 140 kVp with a standard of 120 kVp) emitted from a single source and received by a single detector. The concept of dual-energy computed tomography (DECT) is almost as old as the CT technology itself; DECT initially required substantially higher radiation doses (nearly two times higher than those employed in single-energy CT) and presented problems associated with spatial misregistration of the two different kV image datasets between the two separate acquisitions. The basic principles of single-energy and dual-energy computed tomography and their important physical differences can cause better diagnostic evaluation. Moreover, different organs diagnostic evaluations through single-energy and dual-energy computed tomography have been discussed. According to diverse data and statistics it is controversial to definitely indicate the accurate comparison of image quality and dose amount.

## 1. Introduction

Computerized tomography (CT) has been hailed as a revolutionary diagnostic imaging tool in medicine ever since its introduction in the early 1970s [1]. The arrival of 64-slice CT systems has further validated CT imaging of the heart in clinical routine and also the achieved examination times of 5–10 s lead to short breath hold times, even for patients with dyspnea [2, 3]. There are diverse differences between conventional radiography and computerized tomography. Computerized Tomography (CT) improves upon conventional 2D radiography by producing 3D cross-sectional images of an object from flat X-ray images. The increasing speed of CT along with developments in low-contrast detectability and image quality have allowed the technique to be much more vigorous and this, in turn, has enabled CT to become a majority in medical care throughout the world [4]. Unlike X-ray radiography, the detectors of the CT scanner do not produce an image; they measure the transmission of a thin beam (1–10 mm) of X-rays through a full scan of the body. The image of that section is taken from different angles which allows retrieving the information on the depth (in the third dimension). Conventional or single-energy CT (SECT) uses a single polychromatic X-ray beam (ranging from 70 to 140 kVp with a standard of 120 kVp) emitted from a single source and received by a single detector [5]. The contrast of the image which is caused by this process depends on alterations in photon attenuation of the numerous materials that organize the human body (i.e., soft tissue, air, calcium, and fat) [5]. In SECT, components having diverse fundamental conformations can be characterized by the same CT numbers, making the differentiation and organization of dissimilar types of tissues extremely puzzling. However, the allocation of the scale of grays to each pixel signifies the attenuation of X-rays by the constructions in the tomographic slice [6]. Tissue composition and photon energy level are intrinsic factors and components which can affect the attenuation level of X-ray [5]. Therefore, tissue attenuation can be manipulated by changing photon energy levels which is the fundamental factor of image composition in dual-energy CT [5]. The concept of dual-energy CT was primarily nominated in 1973 [7, 8] and reemerged in the field of clinical radiology with the current technical advances in CT. Although the concept of dual-energy computed tomography (DECT) is almost as old as the CT technology itself, DECT initially required substantially higher radiation doses (nearly two times higher than that employed in single-energy CT) and presented problems associated with spatial misregistration of the two different kV image datasets between the two separate acquisitions [9, 10]. In this regard, dual-energy CT introduced as a first generation dual-source CT system which can develop material variation by using two different X-ray energy spectra [7, 11]. Moreover, two energy levels (typically 80 and 140 kVp) are used to obtain images that can be administered to produce additional datasets and attenuation measurements obtained at a second energy allow the decomposition of a mixture of two or three materials into its constituent materials. Dual-energy CT expands the diagnostic performance and assurance of CT by increasing iodine contrast-to-noise ratio and providing material-specific information [7]. In this regard, the technical diversities and clinical applications of dual-energy CT are ceaselessly on the rise [7, 12]. Overall, rather than increasing image quality and better diagnosis in dual-energy CT, dose reduction is of paramount importance.

This review attempts to discuss the basic and fundamental factors of image formation in single- and dual-energy CT and to compare the image quality and dose in various clinical organs.

## 2. Basic Principles of Image Formation

### 2.1. General Basics According to X-Ray Spectra and Attenuation Coefficient

X-ray photons principally interrelate with matter via the photoelectric effect and Compton scattering producing the diagnostic images used in medicine today. When an atom undertakes the photoelectric effect, the electron from that respected K-shell otherwise referred to as the inner shell is evicted via the incident photon. As that electron is excited, unoccupied space is filled by a neighboring electron, freeing energy as a photoelectron. According to single-energy CT, the attenuation coefficient is counted as the measure of how easily a material can be entered by an X-ray beam. It enumerates how much the beam is “attenuated” (i.e., weakened) by the material it is passing through. A small attenuation coefficient designates that the material in question is relatively translucent, while a larger value indicates greater degrees of opacity. The attenuation coefficient is dependent upon the type of material and the energy of the radiation. Generally, for electromagnetic radiation, the higher the energy of the incident photons and the less dense the material in question, the lower the corresponding attenuation coefficient. In short, when a photon has sufficient energy to overcome the electron's binding energy in the K-shell, that atom undergoes the photoelectric effect. Each substance owns a unique K-shell binding energy, known as the K-edge. There is a significant spike in attenuation that results just beyond the energy of the K-edge; this peak is unique to every material and holds valuable information about the substance's composition. To separate attenuation coefficients into two basis-material components and generate basis-material density images, we must remember that the CT number of water is not energy dependent. Thus CT numbers of soft tissues (with *Z* (eff) values comparable to those of water) remain almost constant when varying the X-ray beam energy. DECT is based on the principle that the attenuation of tissues (reflected by their CT attenuation number in Hounsfield units [HUs]) depends not only on their density but also on their atomic number *Z*, as well as on the energy of the photon beam. To get the best material characterization, DECT should be performed in these conditions [13]:

1. Using two monochromatic (only one X-ray energy) beams with very different energy levels
2. Acquiring both datasets simultaneously
3. Getting images with the same quantity of photons on the detectors

### 2.2. Major Steps in Production of Image

The most sophisticated subjects in dual-energy CT is related to image formation and production during the three fundamental steps: data acquisition, image reconstruction, and image display storage and communication.

#### 2.2.1. Data Acquisition

Currently all systems are based on the rotation of the object and different CT data attainment methods have been advanced essentially [14]. Alternatively, this can be attained by rotating the detector and the source of X-rays around the object, but mathematically both methods are equal [14]. To enhance the scan speed fan beam type or even cone beam geometry and multirow or flat panel detectors which obtain more than one slice during a single rotation are used [14]. The dual CT acquisition system has exceptional technical approaches. Since this early work, a number of technical approaches have been industrialized for obtaining the dual-energy dataset such as sequential acquisition, rapid switching of X-ray tube potential, dual-source CT, and multilayer detector.

Two datasets at diverse tube voltages which are entitled sequential acquisition are an approach that necessitates the least hardware effort [12]. Sequential acquisition can be accomplished either as two subsequent helical scans or as a sequence with subsequent rotations at alternating tube voltages and stepwise table feed [12]. Rapid switching of X-ray tube potential has a significant clinical application which is engrossed on bone densitometry measurements [15]. An indispensable problem which stops the increasing of tube current is the low tube potential measurements which restricts attaining comparable noise levels both low and high to your potential datasets also its alteration in noise limited extension of the technique beyond bone densitometry applications [16]. Dual-source computed tomography (DSCT) is one of the latest innovations in multislice CT technology, which is composed of two X-ray tubes and two corresponding detectors. The gantry rotation time is 0.33 s; the temporal resolution of 83 ms is faster than the other MSCT before [17]. This setup requires nearly twofold investments in hardware but offers important advantages for DECT: the voltage, current, and filter can be chosen autonomously for both tubes to attain an optimal spectral contrast with enough transmission and the least overlap and also the data are developed instantaneously by both orthogonal systems [12]. A fourth mechanism for obtaining dual-energy CT projection data is to use a single high tube potential beam and layered or “sandwich” scintillation detectors. The low-energy data are collected from the front or inmost detector layer, while the high-energy data are collected from the back or outmost detector layer [16].

#### 2.2.2. Image Reconstruction

Image reconstruction involves the use of the attenuation readings collected all around the patient and sent to the computer for processing. The major task of the computer in CT is to reconstruct a digital image using special algorithms to systematically build up the image. One of the more commonplace reconstruction algorithms used in CT is the filtered backprojection algorithm [14, 18, 19]. For optimal clinical benefit, images generated from dual-energy CT datasets should provide structural information similar to that provided by conventional single-energy CT scans; in addition, they can provide material-specific information [20].

To obtain material-specific information, the dual-energy datasets are processed either after the reconstruction of high- and low-energy images (in image-domain decomposition) or before images are reconstructed from high- and low-energy sinograms (in data-domain or projection space decomposition) [20, 21]. There are generally two approaches to extract dual-energy information from projection data. A straightforward method is to subtract equivalent projections and apply filtered backprojection to reconstruct the difference as spectral information and another way is to, first, reconstruct standard CT images consisting of voxels in Hounsfield units and then to use postprocessing algorithms to extract specific spectral information from the difference between the corresponding voxels [12]. Currently, the more commonly used approach is the latter, with the image reconstruction system providing low- and high-kilovoltage images and a series of weighted average images [12].

In addition to reconstruction methods a novel scheme based on measurement of DECT data has been advanced in order to attain clinically satisfactory DECT images from low-mAs acquisitions. In this scheme, enthused by the success of edge-preserving nonlocal means (NLM) filtering in CT imaging and the inherent features underlying DECT images, i.e., global correlation and nonlocal similarity, an averaged image induced NLM-based (aviNLM) regularization is combined into the penalized weighted least-squares (PWLS) framework [22]. More prominently, it provides the best qualitative outcomes with the premium details and the fewest noise-induced artifacts, due to the aviNLM regularization learned from DECT images [22].

Also, to suppress momentous statistical noise in the noisy and inadequate sinograms, an adaptive sinogram restoration (ASR) method is first suggested with attention of the statistical property of sinogram data, and then to additionally obtain a high-quality image, an entire deviation based projection onto convex sets (TV-POCS) method is implemented with a slight alteration [23]. For easiness, the current reconstruction strategy was termed as “ASR-TV-POCS.” Experimental outcomes have confirmed that the current ASR-TV-POCS method can attain promising improvements over other current methods in terms of the noise reduction, contrast-to-noise ratio, and edge detail conservation [23].

Moreover, an innovative adaptive-weighted total variation (AwTV) minimization model for low-dose CT image reconstruction from sparse-view projection measurements needs to be introduced [24]. By presenting an anisotropic diffusion-based adaptive weight to reserve the edge info in the conventional TV minimization paradigm, the gain in modifying the oversmoothing on the edges in the conventional TV minimization was detected by comparing the presentation of the presented AwTV-POCS implementation with the established TV-POCS algorithm [24].

#### 2.2.3. Material Identification

The most major clinical contribution is from iodine and water; new algorithms permit the separation and characterization of supplementary material datasets that have shown multiple potential applications [25]. Various material-specific images gained from a single contrast-enhanced DECT acquisition permit radiation dose reduction by decreasing the need to implement multiphase attainment while providing the essential info for the diagnostic task [25]. The variation of iodine can be observed as a most auspicious application, which can be expected to expand the valuation of vascular malady in CT angiography [11]. Also, an uptake of iodinated contrast material can be determined in a single phase examination more precisely and can develop tissue categorization [11].

#### 2.2.4. Image Display Storage and Communication

PACS has been extensively used in most of large-scale hospitals to obtain and store archived images from medical imaging modalities (including CT, MR, CR, DX, and any other DICOM devices, except US) and dispense them to DICOM devices [26, 27]. DICOM data saved in PACS servers will not deform images. The images can be stored for long periods of time and can be recovered anytime in any computer where PACS client has been set up and also surgeons trained in a few hours can process DICOM by PACS like radiologists and are able to get more beneficial information [26]. There are no differences in the display of dual-energy CT images and all the steps are similar to other CT modalities.

### 2.3. Detector Technology

In SECT most manufacturers share a common detector enterprise; that is, the compressed design has three indispensable layers: conversion of X-ray to light (scintillator), light to current (photodiode), and a substrate to provide the mechanical and electrical infrastructure [28]. Current DECT methodologies either rely on completely separate X-ray sources and corresponding detectors or rely on reading out the projection data at dissimilar time points [12]. Two-layer or “sandwich” detectors with different spectral compassions could offer spectral information in single-source systems but to date have not been employed in clinical scanners [12]. In the future, cadmium-based semiconductors, such as CdZnTe, may serve as semiconductors for photon-counting detectors, which resolve the energy of each individual photon, a method already used in nondestructive material testing and luggage scanners at airports. However, this detector technology cannot yet manage with the high photon flux and cannot supply the high image quality obligatory for clinical CT [12].

### 2.4. Radiation

Although the consequences of preliminary studies showed that dual-energy CT produce higher radiation doses in comparison with single-energy CT [29], many succeeding clinical studies of dual-source dual-energy CT have revealed that dual-energy CT exposes patients to radiation doses similarly to those received during conventional single-energy CT [20, 30, 31]. The outcomes of the more recent studies on dual-energy CT achieved with fast kilovoltage substituting have proved aforementioned result [20].

### 2.5. Clinical Applications of Dual-Energy CT

Dual-energy CT uses can largely be divided into exploration of material-nonspecific and material-specific energy-dependent information [7]. Both assessments can be qualitative or quantitative and the former contains virtual monoenergetic imaging, effective atomic map, and electron density map [7]. Dual-energy CT offers thrilling applications and possibilities that are previously inaccessible with conventional single-energy CT. The potential assistances of DECT comprise increased lesion detection and classification, improved oncologic staging and assessment of treatment reaction, and reduced artifacts, all at comparable or even reduced radiation doses [5].

Assessing the quality and dose variance between SECT and DECT plays a key role in detecting abnormalities. There are diverse clinical applications of dual-energy CT which are being discussed in the following topics, emphasizing the image quality aspects such as CNR, SNR, and dose underpinned by statistical data and diagrams.

#### 2.5.1. Head and Neck

**Figure 1:**
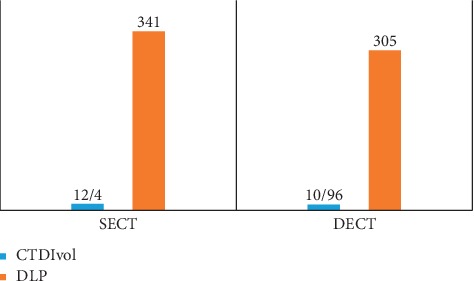
CTDIvol and DLP comparison. The comparison of CTDIvol and DLP factor between patients with the mean age 58 ± 17 years through DECT and SECT in head and neck. All numbers are mean ± SD mGy [32].

**Figure 2:**
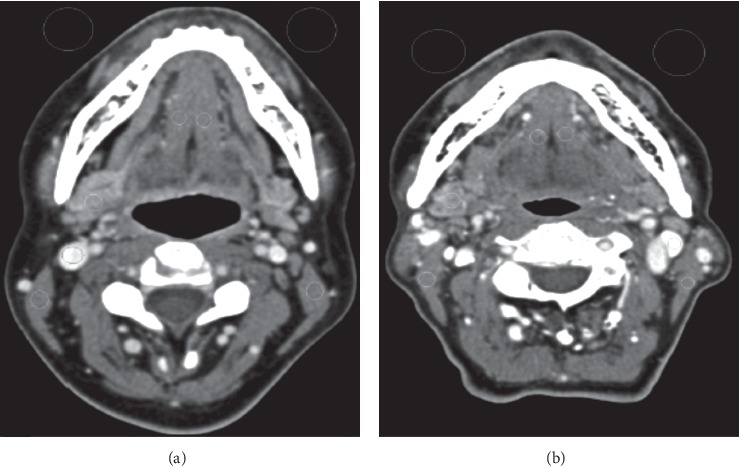
Axial contrast-enhanced images of neck. DE WA images; (a) SE image (b). Both in 120 kv [32].

Head and neck are two of the most indispensable and intrinsic diagnostic parts of the body because of its complicated anatomy and numerous physiological processes. Due to an examination (patients mean age 58 ± 17 years) diverse image parameters have been compared between dual-energy and single-energy CT which are going to be discussed below (Figure 1). The CTDIvol and DLP were significantly lower by 12% and 10%, respectively, in DECT compared with SECT protocols, with no significant differences in noise level, attenuation measurements. In addition to, the subjective image quality between the 2 protocols has no significant differences in image noise, sharpness, or overall image quality observed between DE and SECT of head and nose [32]. As it can be realized from the comparison of axial contrast-enhanced images of neck, one is DECT and the other is SECT which has been done at 120 kv. Dose rate is slightly lower in DECT (Figure 2).

**Figure 3:**
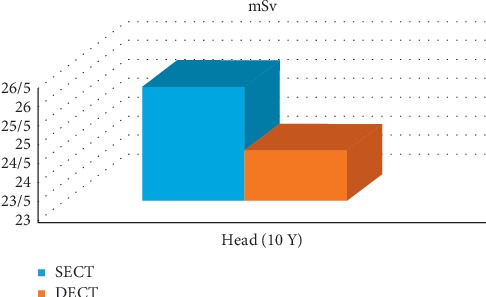
Measurement of CTDIv. CTDIv measurement through head of a 10-year-old patient in both SECT and DECT [33].

Also the similar exam has been done on pediatrics for a 10 year-old child-based phantom in order to examine and compare the CTDIvol of SECT and DECT. The two radiologists who autonomously studied the series of phantom images reported seeing no significant alteration in image quality when images of dual-energy scans were compared with those of single-energy scans [33]. Also CTDIv measurement through organs of a 10-years-old patient is different in both SECT and DECT (Figure 3). It is of note that the amount of dose in SECT is significantly more than DECT [33]. According to diagram CTDIvol of SECT head is 26.01 mSv while in DECT head we reach to the result of 24.33 mSv.

### 2.6. Hepatic Imaging

**Figure 4:**
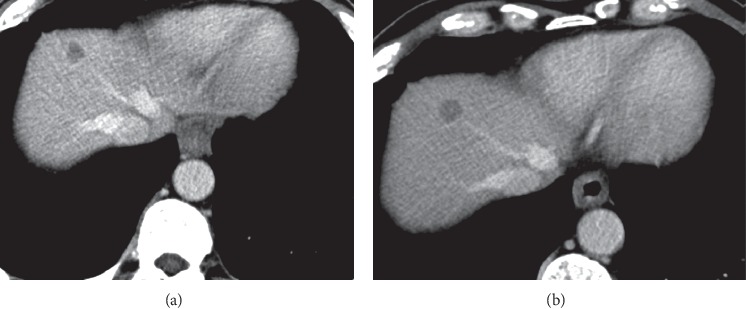
Liver metastasis from gastric carcinoma. SECT scan. (a) CTDIvol = 10.00. (b) DECT scan obtained 57 days after (a) for both mean kv is 90 [34].

**Figure 5:**
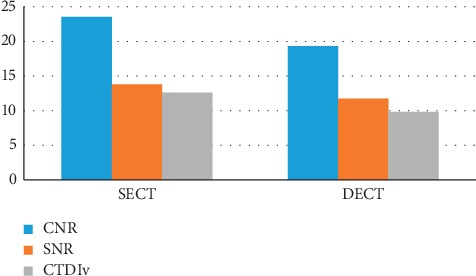
CNR, SNR, and CTDIv. Comparison of CNR, SNR, and CTDIv (mSv) in both SECT and DECT in liver [34].

Liver or hepatic imaging is one of the vital examinations in diagnostic radiology. In contrast to other studies, filtration is the main factor in image dose and quality in hepatic imaging. For DE protocols without added filtration, the blended images have lower CNR than the SE images collected at the optimal kVp without considering the patient size. Furthermore, if tin filtration is applied to the high-energy spectrum, for precontrast scans, the composite CNR of the DE blended image can recover noticeably and surpasses the CNR of SE images without tin filtration [34]. An optimized SE protocol produces up to 5% higher CNR for a range of clinical tasks and a clinical study have observed that 120 kVp SE scans have better image quality than blended DE images which do not have a fundamental CNR advantage over enhanced SE images (Figure 4) [34]. According to Figure 5, three factors (CNR, SNR, and CTDIv) have been examined in liver and a remarkable reduction in all factors is obvious in DECT. As it can be seen in Figure 5, CNR and SNR are 23.55, 19.33 and 13.81, 11.75, respectively, in SECT and DECT in hepatic and CTDIvol in SECT is 12.61 mSv while it is 9.81 mSv in DECT. In short explanation CNR, SNR, and CTDIvol in SECT of hepatic are more than DECT of hepatic.

### 2.7. Abdominal Imaging

**Figure 6:**
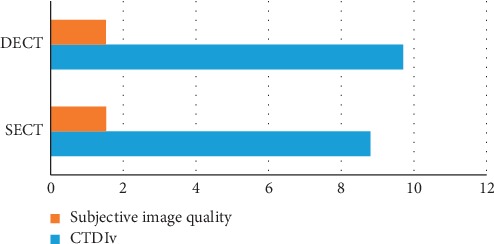
CNR, SNR, and CTDIv. Comparison of CNR, SNR, and CTDIv (mSv) in both SECT and DECT in abdomen [36].

**Figure 7:**
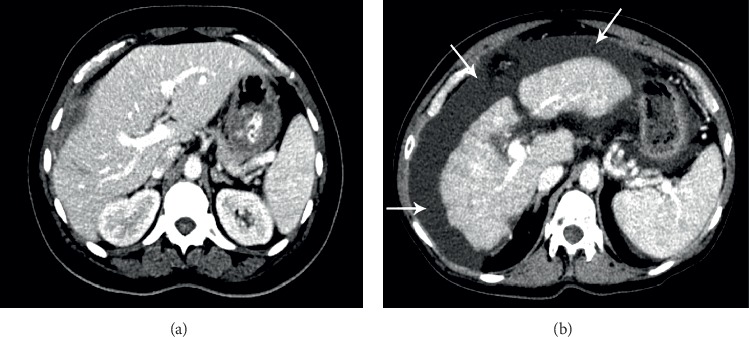
DECT and SECT of abdomen. Abdominal 120 kV second generation. (a) Effective dose adjusted for standard 40 cm acquisition length was 6.5 mSv. Images from a second-generation dual-energy CT (DECT) study in a 58-year-old male with a BMI of 26.5 kg/m^2^ demonstrating extensive ascites (arrows). (b) Adjusted effective dose was 7.0 mSv [36].

The role of dual-energy CT is becoming progressively predominant in abdominal imaging due to the obtainability of scanners and growing field of research. This role of DE in abdominal imaging has been significant in acute bowel ischemia. The addition of iodine maps and 40 keV monoenergetic images to standard single-energy CT images was found to increase reader confidence and precision in diagnosing acute bowel ischemia. Ischemic segments have been found to have lower densities and iodine concentrations in comparison of nonischemic segments [35]. A broad comparison of CNR, SNR, and CTDIv in both single- and dual-energy CT of the abdomen is shown in the diameter below [36]. All the parameters included just mean of CTDIv and subjective image quality. It is necessary to indicate that there are two different ways of measuring image quality, featuring a subjective and an objective approach. According to Figure 6 the factor of subjective image quality of abdominal imaging in DECT is 1.52 and in SECT is 1.53 and also CTDIvol in SECT of abdominal imaging is 8.8 mSv and in DECT is 9.7 mSv. These results indicate that subjective image quality in SECT is more than DECT and also CTDIvol in SECT is less than DECT. Here is a comparison of DECT and SECT of abdomen which is done at 120 kV. As it is transparent, CNR and SNR are better in DECT of abdomen rather than SECT of abdomen (Figure 7).

**Figure 8:**
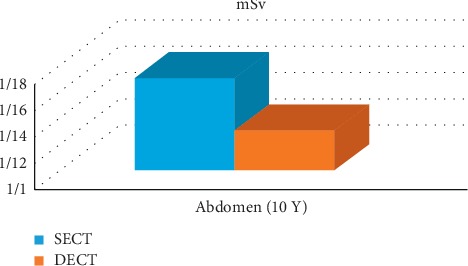
Measurement of CTDIv. CTDIv measurement through abdomen of a 10-year-old patient in both SECT and DECT [33].

To compare the CTDIvol of SECT and DECT, a study has been done on pediatrics for a 10-year-old child phantom (Figure 8) [33].

### 2.8. Urinary Imaging

Dual-energy CT has a number of clinical applications in the assessment of the urinary system particularly in the realm of artifact reduction and material composition.

- Renal stone composition:
- Renal calculi are composed of different substances such as uric acid, calcium phosphate, calcium oxalate, cystine, and brushite. Clinical administration diverges by stone type. Dual-energy CT uses progressive postprocessing techniques to determine the composition of the calculi accurately [37–39]. For example, if a stone is principally fabricated of uric acid, patients can endure standard urinary alkalinization rather than have an interventional process [40].
- Pseudoenhancement of renal cysts:
- Virtual monoenergetic images ranging from 80 to 90 keV created from dual-energy datasets can be fashioned to overcome beam hardening and partial volume of indeterminate renal masses. This can be done retrospectively and act as a convenient utensil when calculating for pseudoenhancement of renal cysts [15, 41].

### 2.9. Virtual Noncontrast Imaging

**Figure 9:**
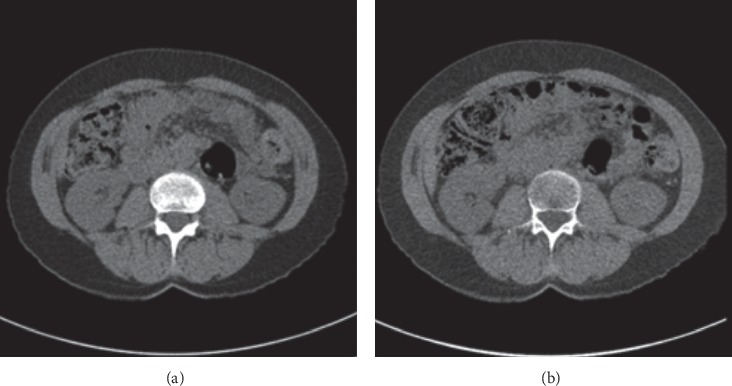
SECT and virtual noncontrast comparison. Single-energy image series before administration of intravenous contrast media. (a) A virtual noncontrast series from the dual-energy contrast-enhanced images. (b) kv used in this examination is 120 [43].

**Figure 10:**
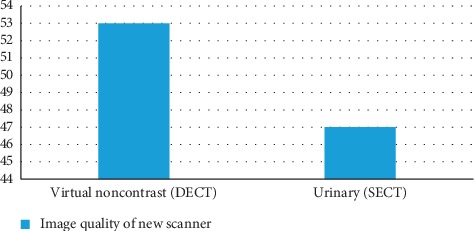
Comparison of image quality between SECT urinary and DECT VNC. It is clear that image quality in DECT virtual noncontrast is greater than SECT urinary [43].

Virtual noncontrast imaging is an image postprocessing technique used to create “noncontrast” images of contrast-enhanced scans via the subtraction of iodine. It is an imaging technique unique to dual-energy CT. Clinical applications of this method have been bolded in differentiation of hemorrhage from iodinated contrast. Contrast staining of the brain parenchyma after iodinated contrast can lead to interpretation issues, especially when the density of contrast is similar to that of blood. Virtual noncontrast imaging is a precise [42] method in differentiating intracranial hemorrhage from iodinated contrast staining following interventional processes such as endovascular clot retrieval. A complete comparison of image quality between single-energy CT urinary and dual-energy CT virtual noncontrast by utilizing new (Somatom definition) and old scanner (Somatom definition flash) has been done for which the results are shown below. By consuming old scanner system, the image quality of urinary (SECT) is more than virtual noncontrast (DECT). Therefore by consuming new scanner system the image quality of virtual noncontrast (DECT) has been enhanced sensationally in comparison of urinary (SECT) [43]. The image quality comparison of DECT virtual noncontrast and SECT urinary is shown in Figure 9, and the factor in image quality of DECT VNC is 53 which is noticeably more than urinary SECT (47). Moreover, comparison of image quality between SECT urinary and DECT VNC shows that image quality in DECT virtual noncontrast is greater than SECT urinary (Figure 10).

### 2.10. CT Angiography

**Figure 11:**
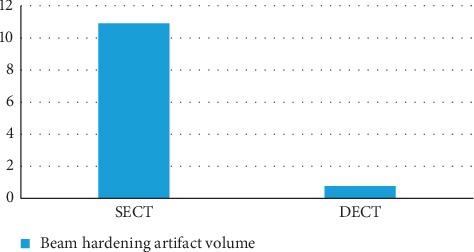
Image quality of DECT and SECT through beam hardening artifact volume. Image quality comparison of surgical group who are undergoing intracranial aneurysm repair with the factor of beam hardening artifact volume and its scale is ml [44].

**Figure 12:**
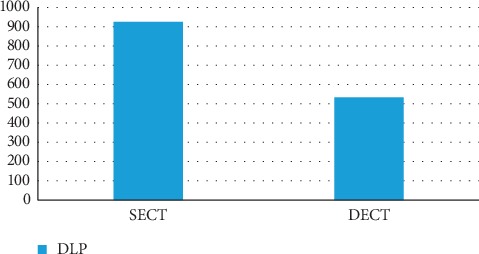
Comparison of DLP in SECT and DECT. Radiation dose comparison of surgical group who are undergoing intracranial aneurysm repair with the factor of DLP and its scale is mGy [44].

**Figure 13:**
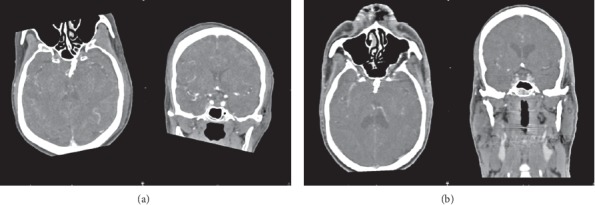
DE-CTA and SE-CTA. Axial and coronal DE-CTA (a) and SE-CTA (b) kv is 100/Sn140 and mAs is 111 [44].

One of the most alluring uses of dual-energy CT is direct CT angiography. In this method, the dual-energy algorithm classifies and eradicates bone, permitting direct imagining of iodinated vessels [16]. On the most recent dual-source CT system, the field of view of the second X-ray source restricts dual-energy bone removal to a 35 cm field of view; single-energy bone elimination algorithms are used outside the 35 cm field of view on that system [16]. The dual-energy technique of CTA has markedly simplified bone removal on cerebrovascular, abdominal, and peripheral run-off studies, replacing a laborious and time-consuming manual chore by an easy step that takes less than a minute to perform on a workstation for image analysis and postprocessing. In addition, based on a newly developed software algorithms, the dual-energy technique allows the automated removal of calcified plaque from the vessel. This permits readily detecting and displaying narrowed segments of the arterial tree otherwise obscured by overlying calcifications without time-consuming manual postprocessing. This technique is particularly helpful in patients with advanced atherosclerosis, when conventional CT angiograms are difficult to interpret. Overall, dual-energy imaging with facilitated bone and plaque removal overcomes limitations of standard CT, improves diagnostic confidence of CT angiographic imaging of the entire vascular territory, and is expected to improve patient care. There are diverse applications of single-energy and dual-energy CT angiography through diverse organs of body. Comparison of image quality and radiation dose in SECT and DECT are shown on Figures 11 and 12. The comparative factor of image quality is beam hardening and the factor of radiation dose is DLP [44] (all these numbers are related to the surgical group who are undergoing intracranial aneurysm repair). In terms of image quality, it is apparent that the factor of beam hardening in SECT (10.91) is noticeably more than DECT (0.77). Here is a comparison of DECT and SECT of brain in both coronal and axial planes. As it is clear, beam hardening artifact is shown in SECT (Figure 13).

Also the DLP factor in surgical group who are undergoing intracranial aneurysm repair in SECT is 925 mGy and in DECT is 534 mGy which shows remarkable DLP in SECT.

### 2.11. Cardiac Imaging

**Figure 14:**
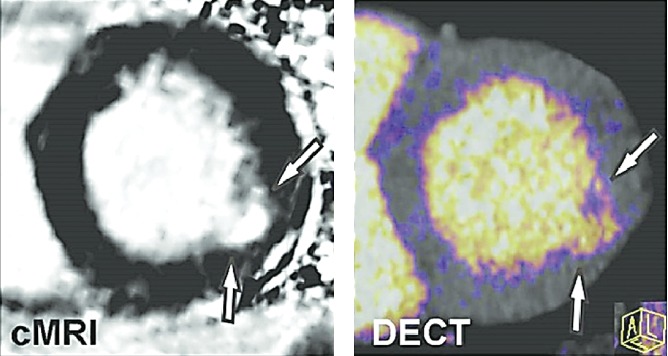
Cardiac magnetic resonance imaging (cMRI) and dual-energy computed tomography (DECT) imaging of myocardial infarction. Iodine color map reconstructed from a DECT image demonstrates good agreement when compared with delayed enhancement cMRI of a subject with prior myocardial infarction of the lateral wall. Similar to cMRI, DECT offers the potential to assess myocardial infarction as well as myocardial salvage through the use of pre- and postcontrast techniques [45].

Coronary computed tomographic (CT) angiography provides angiographic images of coronary stenosis but is a poor predictor of the physiologic importance of any given stenosis. The addition of functional imaging with other modalities such as echo, magnetic resonance imaging which has been compared with DECT in Figure 14, and nuclear medicine is required for patient risk stratification and treatment planning. Dual-energy CT in combination with CT angiography may supplant the need for multiple modality imaging by virtue of its ability to provide for improved plaque characterization and analysis of downstream myocardial perfusion and myocardial viability. Primary proof on the valuation of myocardial ischemia and infarction by DECT as an addition to coronary CT angiography recommend that DECT is an auspicious step toward wide-ranging assessment of coronary heart malady with a single noninvasive modality. However, considerable research examinations will be obligatory before DECT can be considered for repetitive clinical application [46]. Finally, the motion artifacts significantly reduce image quality. However, due to the rapid scanning of dual-energy CT and fast KV switching CT, the motion artifacts are effectively avoided.

## 3. Conclusion

CT and its comprehensive usage have become one of the most indispensable components in medical field especially in the diagnosis of several diseases. SECT and DECT have developed CT diagnostic potentials in several means. In this review article we have discussed the basic principles of single-energy and dual-energy computed tomography and their important physical differences which can cause better diagnostic evaluation. Moreover, different organs diagnostic evaluations through single-energy and dual-energy computed tomography have discussed. According to diverse data and statistics it is controversial to definitely indicate the accurate comparison of image quality and dose amount. In more items single-energy computed tomography has shown better image quality rather than to dual-energy computed tomography also in more items and organs single-energy computer tomography has shown greater CT dose index volume in comparison to dual-energy computed tomography.

## Abbreviations

SECT: Single-energy computed tomography
DECT: Dual-energy computed tomography
DSCT: Dual-source computed tomography
CTDIv: Computed tomography dose index volume
DLP: Dose length product
SNR: Signal-to-noise ratio
CNR: Contrast-to-noise ratio
SE-CTA: Single-energy computed tomography angiography
DE-CTA: Dual-energy computed tomography angiography.
